# Evolution of Genomic Imprinting with Biparental Care: Implications for Prader-Willi and Angelman Syndromes

**DOI:** 10.1371/journal.pbio.0060208

**Published:** 2008-08-26

**Authors:** Francisco Úbeda

**Affiliations:** Department of Ecology and Evolutionary Biology, University of Tennessee, Knoxville, Tennessee, United States of America; Harvard University, United States of America

## Abstract

The term “imprinted gene” refers to genes whose expression is conditioned by their parental origin. Among theories to unravel the evolution of genomic imprinting, the kinship theory prevails as the most widely accepted, because it sheds light on many aspects of the biology of imprinted genes. While most assumptions underlying this theory have not escaped scrutiny, one remains overlooked: mothers are the only source of parental investment in mammals. But, is it reasonable to assume that fathers' contribution of resources is negligible? It is not in some key mammalian orders including humans. In this research, I generalize the kinship theory of genomic imprinting beyond maternal contribution only. In addition to deriving new conditions for the evolution of imprinting, I have found that the same gene may show the opposite pattern of expression when the investment of one parent relative to the investment of the other changes; the reversion, interestingly, does not require that fathers contribute more resources than mothers. This exciting outcome underscores the intimate connection between the kinship theory and the social structure of the organism considered. Finally, the insight gained from my model enabled me to explain the clinical phenotype of Prader-Willi syndrome. This syndrome is caused by the paternal inheritance of a deletion of the PWS/AS cluster of imprinted genes in human Chromosome 15. As such, children suffering from this syndrome exhibit a striking biphasic phenotype characterized by poor sucking and reduced weight before weaning but by voracious appetite and obesity after weaning. Interest in providing an evolutionary explanation to such phenotype is 2-fold. On the one hand, the kinship theory has been doubted as being able to explain the symptoms of patients with Prader-Willi. On the other hand, the post-weaning symptoms remain as one of the primary concern of pediatricians treating children with Prader-Willi. In this research, I reconcile the clinical phenotype of Prader-Willi syndrome with the kinship theory, contending that paternal investment relative to maternal investment increases after weaning. I also propose a genetic composition of the PWS/AS cluster, discuss the effects of new types of mutations, and contemplate the potential side effects of reactivating silent genes for medical purposes.

## Author Summary

Genomic imprinting refers to genes that are silent when maternally inherited but expressed when paternally inherited, or vice versa. Hailed as the most successful evolutionary explanation for genomic imprinting, the kinship theory contends that the paternally inherited copy of a gene, which determines the allocation of maternal resources to her offspring, is selected to extract more resources than its maternally inherited counterpart. The conflict between genes of different parental origin leads to the silencing of one copy but the expression of the other. As originally formulated, the kinship theory assumes that mothers contribute all resources to the raising of offspring. Yet this is not entirely true, as biparental care is common in some mammals, particularly humans. By positing that fathers contribute some resources and analyzing the effect on the kinship theory, I derived new conditions for the evolution of genomic imprinting and discovered that biparental care does not necessarily increase the opportunities for intragenomic conflict. Interestingly, biparental care allows for the evolution of patterns of expression opposite to the ones originally predicted in the kinship theory. Insight gained from my model explains the clinical phenotype of Prader-Willi syndrome, which has so far been a challenge to the kinship theory. Children suffering from this syndrome exhibit a striking biphasic phenotype characterized by poor sucking and reduced weight before weaning but voracious appetite and obesity afterwards. I argue that, in humans, the paternal contribution increases after weaning. This would explain the evolution of genes with the opposite imprinting pattern before and after weaning, and thus sheds light on the bi-phasic phenotype of Prader-Willi syndrome.

## Abstract 3

A familial structure of fathers contributing resources to their offspring may result in a reversal of the expected imprinting pattern and may provide an evolutionary explanation for the striking bi-phasic phenotype of patients with Prader-Willi syndrome.

## Introduction

Imprinted genes violate Mendel's laws by exhibiting an expression conditioned by their parental origin [1]. Either they are silent when maternally inherited (MI) and expressed when paternally inherited (PI), or vice versa [1].

This form of genetic memory captivated the interest of biologists early on. Since its discovery, many theories on the evolution of imprinted genes have been proposed. One of the first theories presents imprinting as an adaptation against ovarian trophoblastic disease [2]. Varmuza and Mann [2] contend that the inactivation of maternally derived genes in oocytes evolved to prevent the development of unfertilized oocytes into ovarian cancer (see [3] for a review of early theories). More recently, Day and Bonduriansky [4] posit that imprinting results from a conflict between genes selected in the opposite sense in each sex. Silencing of the MI copy of a gene is expected to evolve if the selective advantage of that gene through sons is greater than its selective disadvantage through daughters and vice versa. Wolf and Hager [5] claim that imprinting results from the coevolution between genes expressed in the mother and genes expressed in the offspring. Silencing of the PI copy of a gene expressed in the offspring allows the co-adaptation of maternal and offspring traits.

The kinship theory of genomic imprinting (henceforth the kinship theory)—one of the earliest theories on the evolution of imprinting—is currently the most widely accepted [6,7]. The theory's strength lies in its capacity to explain many empirical aspects of the biology of imprinted genes [6–9]. Consider the set of genes expressed in an offspring that influence the allocation of maternal resources. The kinship theory argues that the PI copy of these genes is selected to extract more resources than the MI copy [10]. This is true as long as there is certain asymmetry between matrilines and patrilines, an asymmetry that can be caused by a change in reproductive partners or a male-biased dispersal among others [10]. The kinship theory differentiates two types of gene: those that enhance the allocation of maternal resources when up-regulated, resource enhancers (REs); and those that inhibit the allocation of maternal resources when up-regulated, resource inhibitors (RIs). If the PI copy of an RE is selected for a greater expression than the MI copy, the MI copy will be silenced. If the PI copy of an RI is selected for a lesser expression than the MI copy, the PI copy will be silenced.

The assumptions behind the kinship theory have been intensely scrutinized in the biological literature. One assumption, however, has been largely ignored: mothers are the only source of parental care. In the case of mammals, assuming that fathers contribute little or no resources to their offspring is not always a realistic assumption. Although paternal provision is uncommon among mammals in general (less than 10% of all genera), it is not uncommon in important orders (Perissodactyla, Carnivora, Rodentia, and Primates) (almost 40% of genera within each order) [11]. More importantly, paternal contribution is common in humans [12–17]. Therefore, to understand the evolution of genomic imprinting in mammals, the kinship theory should be extended beyond maternal contribution of resources. I generalized the kinship theory by formulating a model in which both parents can contribute any amount of resources and maternal contribution only is a special case.

I start by discussing the evidence on paternal provision of resources in mammals. Then I elaborate the first model that considers the role of paternal resources in the evolution of genomic imprinting. This model allows me to address two questions: Given biparental care, does genomic imprinting evolve? And, which one of the patterns of expression evolves? In the original formulation of the kinship theory, lifetime monogamy is the only exception to the evolution of imprinting. I find a new condition such that imprinting does not evolve even when there is polygamy. Furthermore, in the original formulation of the kinship theory, for each type of gene, only one pattern of expression is expected to evolve. I conclude that, for some distributions of parental costs, the MI copy of an RE gene becomes silent but, for the rest of the distributions, the PI copy is the one that evolves to be silent. Interestingly, such reversion in the pattern of expression does not require that fathers contribute more resources than mothers. This exciting result illustrates the intimate connection between the expectations of the kinship theory and the social structure of the organism considered.

In the second part of this research, I apply the insight gained from considering paternal contribution of resources to solve one of the challenges to the kinship theory in its original formulation, namely explaining the clinical phenotype of Prader-Willi syndrome (PWS) and Angelman syndrome (AS) [3,18]. Deletion of the PWS/AS cluster of imprinted genes in human Chromosome 15 results in PWS when paternally inherited, but in AS when maternally inherited [1,19]. Children suffering from these syndromes experience feeding, weight, and growth problems, abnormal activity levels, and mental disabilities. The clinical phenotype of PWS children changes dramatically from poor sucking and reduced weight before weaning, to insatiable appetite and obesity after weaning [19–21]. The change is so dramatic that the medical literature describes this syndrome as biphasic [22]. Patients suffering from AS exhibit prolonged sucking—although poorly coordinated—before weaning [20,23] but appetite and weight problems are not constantly present after weaning [19,24].

None of the theories on the evolution of genomic imprinting can explain the clinical phenotype of PWS and AS Syndromes. The kinship theory, however, has shed light on the syndromes' clinical phenotype before weaning [20,25,26]. According to this theory, loss of the PI copy of the PWS/AS cluster implies the loss of all active REs and that PWS children are expected to present a lower than normal acquisition of maternal resources [21]. Loss of the MI copy implies the loss of all active RIs and that AS children are expected to present a greater than normal acquisition of maternal resources [21]. In the original formulation of the kinship theory, no room exists neither for the inversion of the clinical phenotype nor for the symptoms exhibited after weaning by PWS children. Furthermore, it has been pointed out that, after weaning, the clinical phenotype of PWS patients is the opposite to the one predicted by the kinship theory [3,18], which challenges the validity of this theory [3,18].

Haig and Wharton [21] proposed an alternative interpretation of the clinical phenotype that is consistent with the kinship theory. They assume that the more solid food an offspring gets, the less the breast milk it consumes. The substitution of breast milk for solid food would result in a cost to the offspring, because breast milk is of superior quality—both from a nutritional and an immunological perspective—and would result in a benefit to the mother because providing solid food is less costly to her—either because direct provisioning is less costly or because the offspring itself, or other members of the group, contribute to the provision of solid food [21]. If this were the case, a locus controlling appetite for solid food after weaning would be an RE and thus expressed when paternally inherited. Loss of the PI copy would result in greater than normal appetite for solid food and obesity would ensue. It is not clear what type of limitation would result in genes in the offspring being selected to substitute breast milk for solid food as oppose to using solid food as a supplement to breast milk.

In the second part of this research, I contend that the consideration of paternal investment provides a unique insight into the evolution of PWS and AS Syndromes. I reconcile the kinship theory with the clinical phenotype of PWS and AS. I also predict the composition of the PWS/AS cluster of imprinted genes and discuss the evolution of a new type of imprinted gene that exhibits a different pattern of imprint at different moments during development. These results are important not only from a theoretical but also from an applied perspective, as they may contribute to a better understanding of these syndromes. This is important because the frequency of PWS and AS has increased in recent years with the use of assisted reproductive technology [27]. Furthermore, the voracious appetite and obesity exhibited in PWS children after weaning are main concerns of caretakers and pediatricians treating them [19]. The results presented here imply that any attempt to treat these diseases by reactivating silent genes would require considering not only the type of gene reactivated but also when, within developmental time, this gene is expressed. If the time factor is ignored, reactivation could achieve the opposite result to the one expected. These results illustrate how evolutionary theory can have an impact on medicine [28].

## Results

### Paternal Care in Mammals

In this section, I discuss the evidence of paternal care in mammals. I will concentrate on two aspects of paternal care relevant to the arguments discussed in this paper: whether fathers provide food to their nuclear families and whether this contribution increases after weaning.

The term paternal care is used in the biological literature either in a broad or a narrow sense. In a broad sense, paternal care refers to any action of a male that increases the reproductive success of related or unrelated offspring [11]. This is the definition adopted in most studies of natural history of mammals. In a narrow sense, paternal care refers to any action of a father that increases the reproductive success of his offspring at a cost to the reproductive success of his other offspring [29]. This is the definition adopted in studies of parent–offspring conflict and genomic imprinting. In a narrow sense, the male who acts to increase the reproductive success of an offspring must be the father —not any male— and the action must reduce his ability to invest in his other offspring. This distinction will be relevant when discussing the evidence on paternal care in relation to the evolution of genomic imprinting.

Parental care comes in many forms. When the parental action does not benefit the offspring directly, the term “indirect parental care” is used. Examples of indirect parental care in mammals are resource acquisition and defense, shelter construction and maintenance, and food provision to mothers. When the parental action does benefit the offspring directly, the term “direct parental care” is used. Examples of direct parental care before weaning include gestation and lactation, huddling, and cleaning and transportation of the young; after weaning, they include food provision and defense and socialization of young [11]. In mammals, a marked distinction exists between resources that each parent can provide before and after weaning. While mammalian mothers can provide nutrients directly during the gestation and lactation periods, fathers can only provide food directly after weaning.

**Figure 1:**
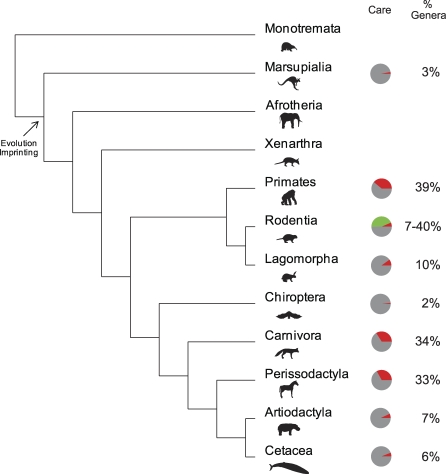
Biparental Care in Mammals The phylogeny of mammalian orders [53] is followed by the percentage of genera showing biparental care [11]. The gray and red colors in the pie charts represent the percentage of genera within the order that exhibit maternal and biparental care respectively. The green color in the order Rodentia corresponds to the percentage of genera expected to exhibit biparental care. While the percentage of genera exhibiting biparental care in mammals as a whole does not exceed 10%, the percentage of genera exhibiting biparental care in Perissodactyla and Carnivora exceeds 30% is close to 40% in primates and is expected to exceed 40% in Rodentia.

Within all mammalian orders, direct paternal care has been observed in 9% of genera [11]. Although this figure suggests that the role of paternal care in the evolution of mammals is negligible, a closer look at its taxonomic distribution provides a very different picture. Direct paternal care has been observed in 33%–34% of genera within the Perissodactyla and Carnivora orders and, more interestingly, in 39% of genera within the Primates [11,30]. Furthermore, it has been suggested that, within the Rodents, the percentage of genera exhibiting paternal care might be greater than any other order; that is at least 40% [11] (Figure 1).

These values should be interpreted cautiously. On the one hand, they are conservative in the sense that the natural history of many mammals is unknown, and the lack of observations on particular genera was recorded as absence of paternal care. On the other hand, these observations correspond to paternal care in the broad sense, and some of them may not qualify as paternal care in the narrow sense. I will further elaborate on the existing evidence on paternal care by discussing the provision of food in two species of non-human mammals—chosen on the basis of data availability— and in human hunter-gatherer societies.

#### Non-human mammals.

*Wolves*. A pack of wolves (Canis lupus) is formed by a breeding pair and their descendants under the age of 3 y [31]. Members of the pack hunt individually for small prey (hares) but together for bigger prey (musk ox and caribou). Weaning starts 35–40 d after birth. Before weaning, the mother provides milk for her pups, whereas the father provides food to his partner. After weaning, both the mother and father provide meat to their pups. Parents either carry small prey or regurgitate meat from bigger prey. Regurgitation often follows from the pup's demand [32] when the pup pokes its muzzle around the parent's mouth (“lick-up”). The father continues providing food for his partner after weaning [32].

Studies on food transfer via regurgitation in arctic wolves show that both mothers and fathers regurgitate to their offspring. In addition, fathers regurgitate to their partners but half as often as they regurgitate to their offspring [32,33]. The total amount of food transferred to the young by each of the parents is the same [32,33]. It is important to notice that the father regurgitates to the mother in the first half of the summer as often as in the second half [32,33]. However, he regurgitates to his offspring four times as often in the second half of the summer than in the first half [33]. Mid-summer (45 d into the summer) occurs a little after the pups, born in late spring, are weaned (35–40 d after birth). These data suggest that paternal food transfer increases dramatically after weaning: indirect care (via the mother) is maintained at the same level before and after weaning but, in addition, after weaning, direct care is provided and occurs four times more often than indirect care.

Studies on den attendance indicate that, early in the breeding season, mothers rarely abandon their den, while the fathers spend most of their time away from the den, hunting and resting [32,34]. The sex difference in den attendance declines progressively until there is no difference between the sexes, which happens at weaning [32,34]. Thurston [34] argues that the time spent by each parent with the den is directly related to the amount of begging for food that each parent is exposed to. If this is true, before weaning, the father limits his exposure to the mother's begging while the mother attends to the pups' demand for milk. After weaning, the father increments his exposure to begging from the mother and his pups while the mother limits her exposure to pups' begging as she spends more time hunting away from the den. Thurston [34] contends that this data provides evidence of symmetric biparental care (in terms of food transfer) after weaning. It also provides evidence of a reduction in the amount of food transferred by the mother after weaning and an increment in the amount of paternal care after weaning.

*Owl monkeys.* A family of owl monkeys (Aotus azarai) is usually formed by a breeding pair and their descendants under the age of 4 y. Weaning starts 4 wk after birth [35]. Before weaning, the mother provides milk to her offspring. After weaning, both the mother and the father start providing leaves, flowers, and fruits of fig and guazuma trees to their younglings [35]. Food transfer follows the offspring's demand when it reaches out its hand toward a potential donor while moving its mouth towards the food object in which it is interested (“beg”). Interestingly, mothers provide food to their mates and not the other way around [35]. Maternal provision of food to fathers happens during gestation when the offspring cannot benefit from a greater food intake of the father. This has been interpreted as evidence that resource transfer between breeding pairs responds more to pair bonding than indirect provision of parental care to offspring [35].

Owl monkey fathers may transfer food to their offspring three times as often as mothers do [35]. However, there is variability among primates in this respect; marmoset fathers (Callithirix flaviceps) transfer food to their young as often as mothers do [36]; tamarin mothers (Saguinus mystax) transfer food to their young more than seven times as often as fathers do [37]. In the studies of owl monkeys considered, the paternity of the male donor was not confirmed and the male donor might be an unrelated male. However, if the breeding male was replaced by another, the stepfather would transfer food to related but not unrelated offspring [35]. The rates of begging in offspring and food transfer from fathers increase significantly when the offspring start weaning [35]. During the weaning period, owl monkeys experience high growth rates, rates that are sustained by the food that fathers provide [35].

These data suggest that paternal food transfer increases significantly after weaning. There is no indirect provision of food to the offspring. Direct provision of food is made entirely by the mother before weaning, and mostly (75% of it) by the father after weaning.

#### Humans.

The only insight on parental care in ancestral human societies comes from the anthropological literature on modern hunter-gatherer societies. Such evidence should be read cautiously, as the behavior of modern hunter-gatherer societies may have diverged considerably from the behavior of ancestral human societies. A group of hunter-gatherers is formed by several reproductive couples and their offspring under the age of 16 y [17]. Men produce more food than women, and children's production is almost insignificant [38]. There are also important differences in the type of food provided by each sex. Males provide the vast majority of meat (protein), while women provide the majority of roots and fruits [38]. Young contribute mostly fruits [38].

The food produced by adult males is either contributed to their nuclear families or shared with the rest of the group. Hunter-gatherer men often keep small game—and other easy-to-hide resources—for household consumption. This is the case among Meriam men from Melanesia who collect marine turtles [13,16]. Kung men from Botswana hunt geckos and collect baobab fruit for their nuclear family's consumption [12]. Hadza men from Tanzania hunt bush babies and collect honey that they contribute to their households [15]. While hunter-gatherer men tend to share big game with members of the group other than their nuclear families, they do keep part of the catch for household consumption. This is the case among Lamaleras from Indonesia, where the harpooner gets a bigger share of any sperm whale or giant ray hunted [16]. Among Ache men from Paraguay, the hunter gets a bigger share of any armadillo or capuchin monkey killed [14,16]. A third of all food acquired by Hiwi men from Venezuela is kept by the nuclear family of the acquirer [39]. Within the nuclear family of hunter-gatherers, fathers contribute resources to their partners (before and after weaning) and to their children (after weaning) [17]. I am not aware of any data quantifying differences in paternal contribution before and after weaning.

#### Relative contribution after weaning.

In the above examples, I discussed two different scenarios in which the father's contribution increases after weaning. (1) In the owl monkey, fathers do not transfer food to their partners, but they do provide food to their offspring. This is not an isolated case; fathers do not provide food to their partners in lions (Panthera leo) and tenrecs (Tenrec ecaudatus), among others [11]. Because offspring's provision can only happen when the weaning process starts, there is necessarily an increment in the contribution of paternal resources after weaning. This would also be the case when the paternal contribution to the mother is negligible compared to the paternal contribution to the offspring. (2) In the wolf, fathers transfer to their partners the same amount of food before and after weaning and contribute food to their offspring after weaning. Because indirect paternal care remains constant whereas direct paternal care is provided only after weaning, there is an increment in the relative contribution of paternal resources after weaning if all other variables remain equal.

Besides evidence discussed here, there are two reasons why one would expect an increment in paternal care relative to maternal care after weaning. The first has to do with the more intimate relation between mammalian mothers and offspring during gestation and lactation. As a result, it is more likely that before weaning, the mother would meet any demand for food by her offspring. Before weaning, the ability of an offspring to influence the provision of food made by the father is limited. It is difficult to think of any way of influencing paternal provision that is not mediated by the mother. This is not the case after weaning. The second reason why an increment in paternal care would be expected is that even if the offspring is able to influence paternal provision of food to its mother, indirect paternal care enables mothers to keep part or all of the paternal investment for herself. After weaning, direct paternal food transfer does not leave room for the mother to manipulate paternal investment.

### A Model of Biparental Care

In this section, I generalize the kinship theory [40] by allowing the expression of a gene in an offspring to affect both maternal and paternal investment (as opposed to maternal investment only). The model here presented (see the Methods section for a detailed formulation) relates to a class of models formulated to study either parent-offspring conflict [29,41,42] or the kinship theory [40]. All these models assume that parental care is limited to maternal care, except a model for parent-offspring conflict formulated by Parker [43].

Consider a nuclear family formed by a mother, a father, and their offspring. Both parents have a limited amount of resources to invest either in the current or future offspring. Consider a gene expressed in the current offspring that affects the amount of resources provided by the mother and the father to the current offspring. In particular, a greater expression of the locus containing this gene results in a greater investment of both parents—as opposed to a reduced provision of resources.

Consider a mutant that modifies the expression of either the MI or the PI copy of an RE. The change in expression at this locus results in a benefit *B_O_* to the current offspring at a cost to its parents, *C_M_*, *C_P_*. The parental cost can be subdivided into the cost derived from a greater investment, *C_Mi_*, *C_Pi_*, and the investment elicited by a greater expression, *I_Mx_*, *I_Px_*, that is *C_M_* = *C_Mi_I_Mx_*, *C_P_* = *C_Pi_I_Px_*. Hence, I will talk about maternal *C_Mi_* and paternal *C_Pi_* cost of investment and about maternal *C_M_* and paternal *C_P_* cost of expression. Benefits and costs refer to changes in the number of offspring of the current offspring, and in the number of grand offspring of the current offspring's parents respectively.

The extent to which the costs that the father incurs translate into maternal costs, κ*_M_*, and the costs that the mother incurs translate into paternal costs, κ*_P_*, are determined by the mating system of the population. For example, if there is lifetime true monogamy κ*_M_* = κ*_P_* = 1. If mothers die before fathers and fathers replace their partners κ*_M_* ≤ 1 and κ*_P_* = 1 with κ*_M_* being smaller as the cost of finding a new partner becomes smaller. If parents change partners for the production of every offspring, κ*_M_* = κ*_P_* = 0. In theory, it is possible that κ*_M_*, κ*_P_* > 1 . However, the biological conditions for this happening are rare (see discussion in [44]) and will be ignored in this research, that is, 0 ≤ κ*_M_*, κ*_P_* ≤ 1.

Consider a population in which the resident gene shows the same level of expression when maternally and paternally inherited. The fate of a rare mutant that, when maternally inherited, increments the expression of the RE locus considered is determined by its inclusive fitness effect:

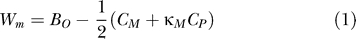



The inclusive fitness effect of a mutant acting on the MI copy is given by the difference between the benefit experienced by the current offspring and half of the cost that the mother incurs. Such cost has two components: the maternal cost of expression, *C_M_*, and the paternal cost of expression, *C_P_*, multiplied by the extent in which costs borne by the father translate into maternal costs, κ*_M_*. While benefits are weighed by the probability that the allele expressed in the current offspring is present in itself, namely one, costs are weighed by the probability that the allele expressed in the current offspring is present in its siblings, namely half.

The fate of a rare mutant that, when paternally inherited, increments the expression of the RE locus considered is determined by its inclusive fitness effect:

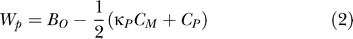



The inclusive fitness effect of a mutant acting on the PI copy is given by the difference between the benefit experienced by the current offspring and half of the cost that the father incurs Such cost has two components: the cost the father incurs due to enhanced paternal investment elicited by up-regulated expression, *C_P_*, and the cost the mother incurs due to enhanced maternal investment elicited by up-regulated expression, *C_M_*, multiplied by the extent in which costs borne by the mother translate into paternal costs, κ*_P_*.

Let the ordered pair 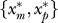 correspond to the level of expression of the MI and PI copies of the allele fixed in the population and x^*^ correspond to the combined level of expression of both copies. The optimal level of expression from the perspective of the MI copy 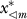 can be derived from imposing condition *W_m_* = 0 in Equation 1. The optimal level of expression from the perspective of the PI copy 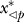 can be derived from imposing condition *W_p_* = 0 in Equation 2.

When 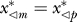 , there is no conflict between the MI and PI copies and imprinting does not evolve .When 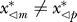 , there is conflict and imprinting does evolve. If 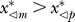 , the intralocus conflict results in the evolutionarily stable pattern of expression 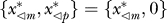 . If 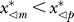 , the intralocus conflict results in the evolutionarily stable pattern of expression 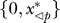 . The direction of the imprint—either MI copy silent or PI copy silent—is neatly summarized by the “loudest voice prevails” principle [10]: natural selection favors silencing of the copy whose optimal level of expression is lower, and expression, at its optimal level, of the allele whose optimal level of expression is greater [10].

The difference between the inclusive fitness effect of the maternally and paternally inherited copies

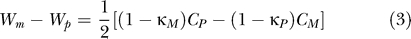
characterizes not only whether there is intralocus conflict but also, in case of conflict, what will be the evolutionarily stable pattern of expression. If *W_m_* − *W_p_* = 0, then 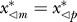 and there is intralocus conflict. If *W_m_* − *W_p_* > 0, then 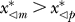 and silencing of the paternally inherited copy evolves. If *W_m_* − *W_p_* < 0, then 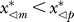 and silencing of the maternally inherited copy evolves.

When paternal investment is negligible, *C_P_* ≈ 0 , silencing of the maternally inherited copy 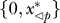 evolves unless all maternal offspring are sired by the same father κ*_P_* = 1. This is the condition derived by Haig [10]. When maternal investment is negligible, *C_M_* ≈ 0, silencing of the paternally inherited copy 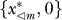 evolves unless all paternal offspring are born to the same mother κ*_M_* = 1.

**Figure 2:**
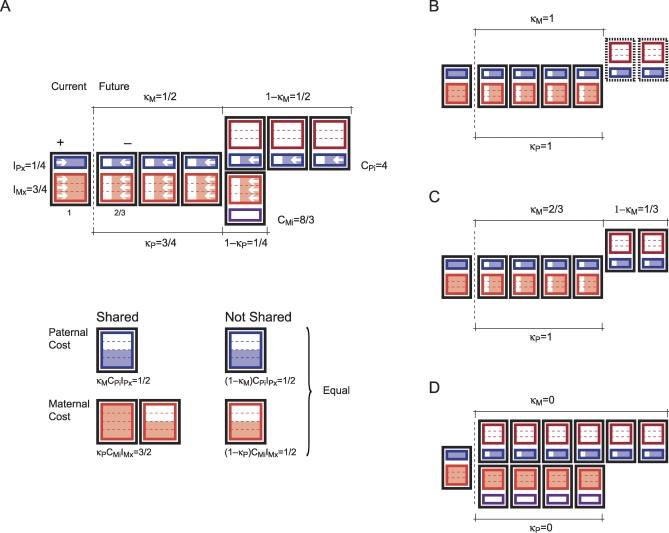
Mating System and Cost Distribution Each rectangle represents an offspring; the two rectangles contained in each offspring represent the parents—mother in red and father in blue—that produced the offspring and the fraction of resources contributed by each parent. The fraction of resources contributed by the mother is σ. The first rectangle corresponds to the current offspring and the following rectangles correspond to future offspring. If the future offspring is aligned with the current one—and carries the same color combination—then it has the same parents as the current offspring. If the future offspring is not aligned with the current one—and carries a different color combination—then it either has a different mother if it is above the current one or has a different father if it is below the current one. Any investment in the current offspring translates into a cost to the father and the mother in terms of residual reproductive value. In this example, the cost experienced by the father is also suffered by the mother with probability κ*_P_* and the cost experienced by the mother is also suffered by the father with probability κ*_M_*. The cost of providing an additional unit of investment is different for the father *C_Pi_* and the mother *C_Mi_*. In case **A**, mothers contribute more than fathers, σ = ¾, the maternal costs shared by the father affects to κ_*M*_ = ½ of the father's residual reproductive value, the paternal cost shared by the mother affects κ_*P*_ = ¾ of the mother's residual reproductive value. The paternal cost for each unit of investment is almost two times the maternal cost. In this scenario, the paternal cost not shared with the mother is equal to the maternal cost not shared with the father. In case **B,** there is lifetime monogamy and when the mother dies, the father does not produce any more offspring even if he had resources to do so. In case **C,** there is sequential monogamy and when the mother dies, the father finds a new mate. In case **D,** father and mother change partners to produce every single offspring.

When both paternal and maternal investment are non-negligible, genomic imprinting does not evolve when there is lifetime true monogamy κ*_M_* = κ*_P_* = 1, and when the paternal cost of expression that is exclusive to the father (not shared by the mother) is equal to the maternal cost of expression that is exclusive to the mother (not shared by the father),

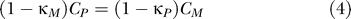
(Figure 2). More interestingly, both patterns of imprint—MI copy silent and PI copy silent—can evolve. If the paternal cost of expression that is exclusive to the father is greater than the maternal cost of expression that is exclusive to the mother, (1 − κ*_M_*)*C_P_* > (1 − κ*_P_*)*C_M_*, then silencing of the PI copy evolves. However, if the maternal cost of expression that is exclusive to the mother is greater than the paternal cost of expression that is exclusive to the father, (1 − κ*_M_*)*C_P_* < (1 − κ*_P_*)*C_M_*, then silencing of the MI copy evolves.

**Figure 3:**
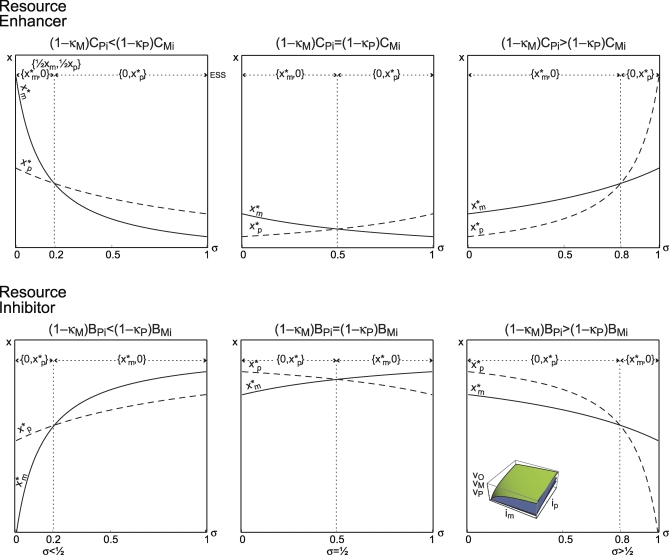
ESS Pattern of Expression The horizontal axis represents the fraction of resources contributed by the mother σ. The vertical axis represents the total level of expression of an RE (top row) and an RI (bottom row). The continuous line corresponds to the optimal level of expression from the perspective of the maternally inherited allele 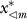 as a function of the fraction of resources contributed by mothers σ. The discontinuous line corresponds to the optimal level of expression from the perspective of the paternally inherited allele 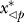 as a function of the fraction of resources contributed by mothers σ. The intersection of both these lines corresponds to the value σ in which the conflict becomes extinct, σ̂. In the top part of each figure, the evolutionary stable pattern of expression 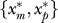 is represented. If (1−*κ_M_*)*C_Pi_* < (1−*κ_P_*)*C_Mi_* (first column) the conflict becomes extinct when mothers contribute less resources than fathers, *σ* < ½. If (1−*κ_M_*)*C_Pi_* = (1−*κ_P_*)*C_Mi_* (second column) the conflict becomes extinct when mothers contribute as many resources as fathers, *σ* = ½. If (1−*κ_M_*)*C_Pi_* > (1−*κ_P_*)*C_Mi_* (third column) the conflict becomes extinct when mothers contribute more resources than fathers, *σ* > ½. The figure inserted at the bottom right corner represents the offspring fitness function *v_O_* (in green) and the parental fitness functions (maternal *v_M_* and paternal *v_P_*) (in gray) used to elaborate the figure, where *v_o_* = log(1 + *i_M_* + *i_P_*), *v_M_* = *z_M_i_M_* + *κ_M_z_P_i_P_*, and *v_P_* = *κ_P_ z_M_ i_M_* + *z_P_i_P_*) where *z_M_* and *z_P_* are constants.

Assuming that the fraction of nutrients provided by the mother to the current offspring is a constant σ, it is possible to determine the critical fraction of resources that contributed by the mother would result in the extinction of the intralocus conflict

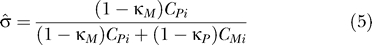
where *C_Mi_* and *C_Pi_* are the maternal and paternal costs of investment in the current offspring. Using the language of the “loudest voice prevails” for values of σ lower than σ̂) σ̂, the maternal voice within the offspring speaks louder than the paternal voice, and silencing of the PI copy evolves. For values of σ greater than σ̂)_,_ the maternal voice within the offspring speaks softer than the paternal voice, and silencing of the MI copy evolves (Figure 3).

It is worth noticing that the extinction of the intralocus conflict does not require that both parents contribute the same amount of resources. More interestingly, the intralocus conflict becomes extinct when the mother contributes more resources than father, σ̂ > ½, if the paternal cost of investment that is exclusive to the father is greater than the maternal cost of investment that is exclusive to the mother,

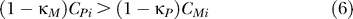
(Figure 3). This might be of particular interest, because in nature, it might be found that mothers contribute more resources than fathers in many cases.

If a greater expression of the gene considered results in a lower provision of resources, symmetric results can be derived. The only difference being that the current offspring incurs in a cost (*C_O_* = −*B_O_*) , while its parents experience a benefit (*B_M_* = −*C_M_*, *B_P_* = −*C_P_*). The expected patterns of expression are opposite to the ones derived for an RE. If the paternal benefit of expression that is exclusive to the father is greater than the maternal cost of expression that is exclusive to the mother, (1 − κ*_M_*)*B_P_* > (1 − κ*_P_*)*B_M_*, then silencing of the MI copy evolves. However, if the maternal cost of expression that is exclusive to the mother is greater than the paternal cost of expression that is exclusive to the father, (1 − κ*_M_*)*B_P_* < (1 − κ*_P_*)*B_M_*, then silencing of the PI copy evolves.

#### Paternal investment changes over time.

Consider the case that the relative contribution of fathers increases after weaning, σ^a^, above a critical value, that is σ^b^ > σ̂ > σ^a^ Consider two possible scenarios (see the Methods section for the full derivation).

**Figure 4:**
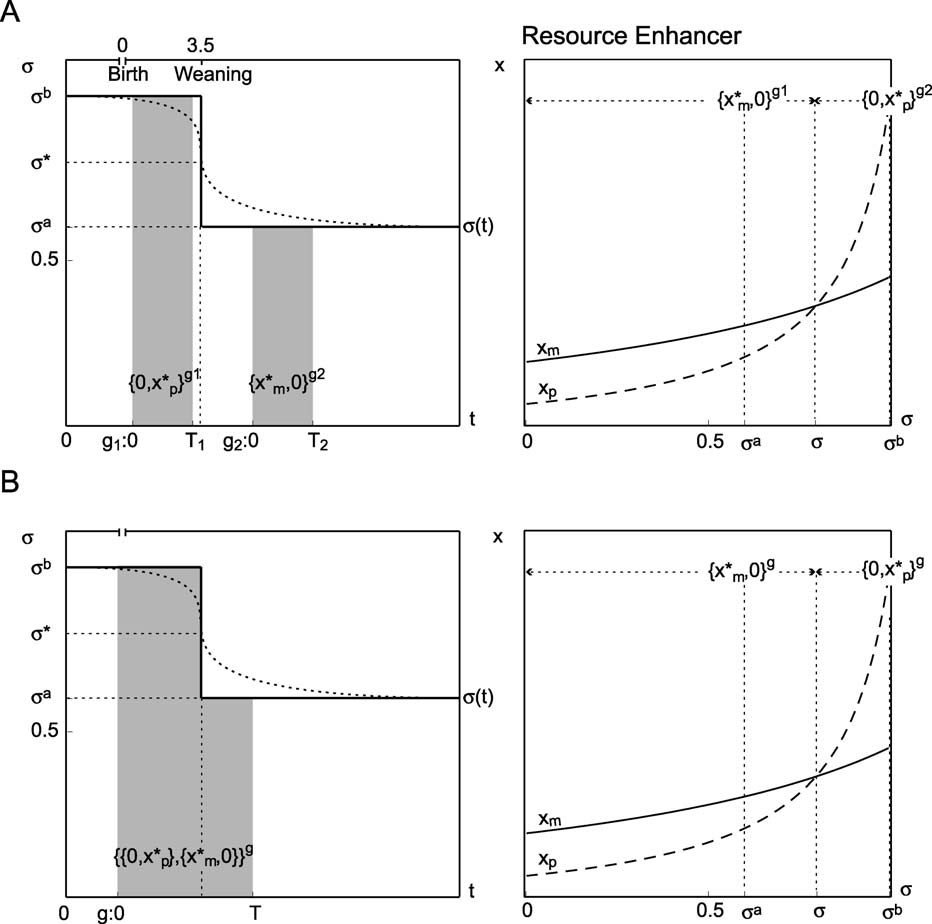
Contribution of Resources as Function of Time I present two pairs of figures. The horizontal axis corresponding to the first figure in each pair represents time, *t*, in the life of an individual. The window of expression of gene *g* occurs from 0 to *T*. The vertical axis represents the fraction of resources contributed by the mother σ. The dotted line corresponds to the more realistic case when the fraction of maternal resources changes continuously from little paternal contribution before weaning to greater paternal contribution afterwards. The continuous line corresponds to a two-period scenario when mothers contribute σ^*b*^ > σ̂ before weaning and σ^*a*^ < σ̂ afterwards. The second figure corresponds to the top right graphic from Figure 3 where the fraction of resources contributed by the mother has been specified. Case **A** corresponds to genes expressed before *g*_1_ and after *g*_2_ weaning. The ESS expression is 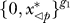 if *g*_1_ expressed before weaning and 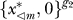 if *g* expressed after weaning. Case **B** corresponds to a gene expressed before and after weaning that can adjust its level of expression. The ESS expression is 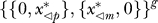 .

(a) There are two genes: one, *g*_1_, expressed before weaning and another, *g*_2_, expressed after weaning. The gene expressed before weaning will be maternally silent 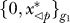 but the gene expressed afterwards will be paternally silent 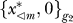 (Figure 4).

(b) There is only one gene, *g*, expressed before and after weaning. Assume that this gene can modify its level of expression in each period of time. Let the two ordered pairs 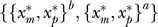 represent the level of expression of the MI and PI copies before and after weaning respectively. This gene will be maternally silent before weaning and paternally silent afterwards 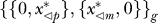 (Figure 4).

If a gene is expressed during a window of time when the paternal investment relative to the maternal investment changes, then this gene will be expected to reverse its pattern of imprint over time. I will use the term “flip-flop imprinted genes” to refer to this type of genes. Flip-flop imprinted genes may achieve a reversion in their pattern of imprint by using different promoters with reverse patterns of imprint during different moments in time.

Notice that the same change in expectations of expression could be caused by changes in *C_Pi_* relative to *C_Mi_* over time. This could be motivated by each parent providing different types of resources with different cost of getting each one over time. For example, mothers switch from providing milk to providing fruits and roots after weaning.

### Kinship Theory and PWS

#### PWS: A challenge for kinship theory.

One of the areas in which the kinship theory has been most successful is in explaining the clinical phenotypes of many diseases linked to imprinted genes [20,26,45]. However the kinship theory faces some difficulties in explaining the clinical phenotype of PWS and AS [3,18].

**Figure 5:**
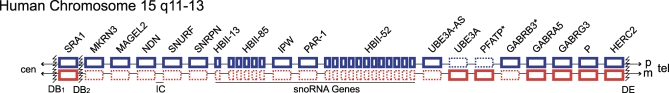
Human PWS/AS Cluster of Imprinted Genes The colors red and blue represent the maternally and paternally inherited alleles. Continuous and dashed lines represent expressed and silent genes respectively.

PWS and AS are caused by the deletion of region q11–13 in human Chromosome 15 [19]. This region contains a cluster of imprinted genes (Figure 5), some of them maternally silent and others paternally silent [46]. Children that inherit the deletion in Chromosome 15 from fathers experience PWS, but children that inherit this deletion from mothers experience AS [19]. PWS and AS can also be caused by uniparental disomy of Chromosome 15 in humans [19], but I will frame the discussion in terms of deletion of region q11–13.

According to the kinship theory, lacking the PI strand of a cluster of imprinted genes will result in the loss of the active copies of any RE and the silent copies of any RI. The remaining strand contains silent REs and expressed RIs. Therefore, the kinship theory predicts that children with PWS will present a clinical phenotype associated with lesser demand of resources. On the other hand, the deletion of the MI strand of a cluster of imprinted genes will result in the loss of the silent copies of any RE and the expressed copies of any RI. The remaining copy contains active REs and silent RIs. Therefore, the kinship theory predicts that children with AS will present a clinical phenotype associated to greater demand of resources.

Children with PWS and AS experience disorders related to eating, appetite, and weight; activity levels; and mental disabilities (see [19] for a comprehensive description of the clinical phenotype). It is the eating, appetite, and weight disorders that are concerning to pediatricians treating patients with PWS and AS [19]. Their interpretation in terms of resource acquisition is fairly straightforward, while in the case of mental disorders, the interpretation is more elusive [47].

**Figure 6:**
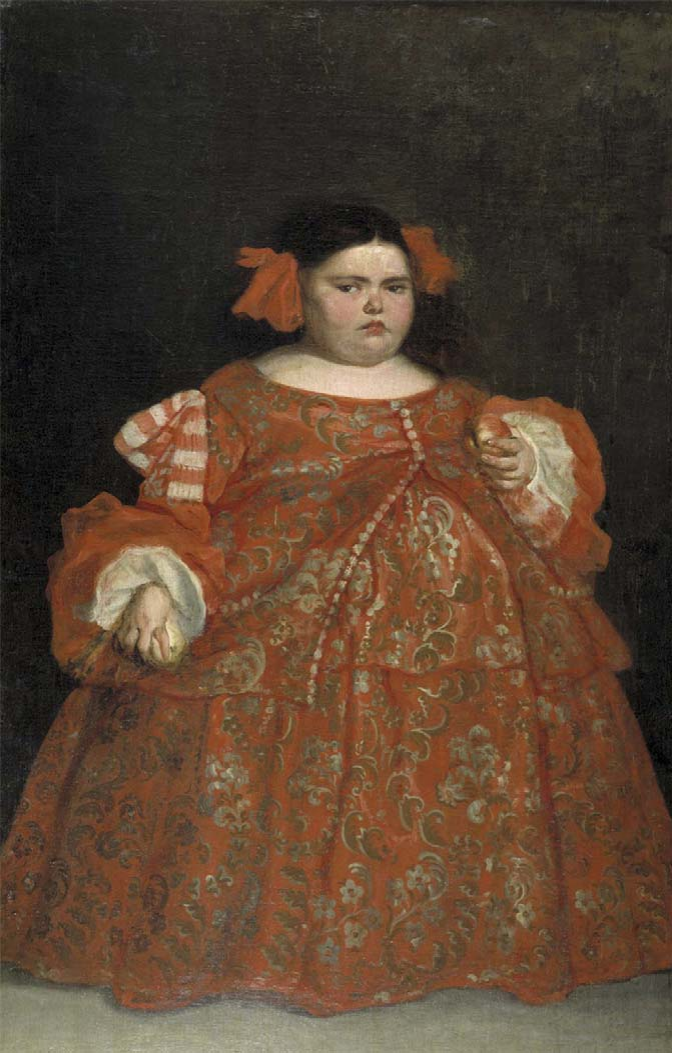
Portrait of Eugenia Martinez Vallejo at Museo del Prado (Madrid) Eugenia Martinez Vallejo was portrayed by Spanish painter Juan Carreño Miranda in 1680. It has been suggested that she had PWS [19]. At the time of the painting, she was 6 years old and in the hyperphagic (over eating) phase of the disease, which occurs after weaning. She weighed 120 pounds (∼54 kg) and was portrayed with two pieces of food in her hands, which correspond to these patients' voracious appetite. Other symptoms pointing toward this disease include her short stature, almond-shaped eyes, small triangular mouth, and small hands.

The clinical phenotype of children with PWS changes dramatically from poor sucking and reduced weight before weaning, to insatiable appetite and obesity after weaning [10–21] (Figure 6). Children with AS, however, spend prolonged periods of time suckling before weaning even though sucking is poorly coordinated [20,23]. After weaning, appetite and weight problems are neither always present nor consistent in one way or the other—some children are fussy eaters but others would do anything to get food [19,24]. The clinical phenotype of children with PWS and AS before weaning meets the predictions of the kinship theory but the clinical phenotype of these children after weaning poses a challenge to the kinship theory [3,18,21].

One way to explain the voracious appetite observed after weaning is as a by-product of the reduced appetite before weaning with little or no adaptive explanation [20]. More interestingly, Haig and Wharton [21] suggest that the post-weaning appetite might be consistent with the kinship theory. They notice that the dietary requirements of an offspring shifts from breast milk before weaning to a combination of breast milk and solid food afterward. Haig and Wharton [21] argue that if: (a) a reduction in solid food intake is compensated by an increment in breast milk consumption, (b) breast milk is superior to solid food (nutritionally or immunologically), and (c) provision of solid food is less costly to the mother (either because direct provisioning is less costly or because mothers are not the only providers of solid food), then, according to the kinship theory, a gene that determines the appetite for solid food is an RE and would evolve to be silent when MI and expressed when PI. Consequently, loss of the PI copy would result in a greater than normal appetite after weaning, which is consistent with the clinical phenotype of children with PWS. The bi-phasic phenotype would be consistent with the kinship theory if there are two genes: (1) an RE that controls consumption of breast milk and is expressed before weaning, and (2) another gene whose enhanced expression reduces appetite for solid food and is expressed after weaning.

The transition from a diet based on the mother's breast milk only to a diet that incorporates solid food provided only partially by the mother plays an important role in the reversion of the clinical phenotype of children with PWS. But what is the role played by solid food? Haig and Wharton [21] assume that intake of solid food and consumption of breast milk are mutually exclusive. While this is certainly possible, it is not clear how access to breast milk will be conditioned by the amount of solid food consumed by the offspring. Furthermore, the more solid food comes from sources other than the mother, the less likely that such conditioning exists or is enforced. Haig and Wharton [21] argue that provision of solid food is less costly to the mother, because part of this food might be gathered by the offspring or contributed by the father or other members of the group. If it is provided by the father—or members of the group closely related through the patriline—then it is necessary to consider explicitly the father's contribution of resources.

##### Reconciling kinship theory and PWS.

I suggest a new way to reconcile the clinical phenotypes of PWS and AS with the kinship theory. My hypothesis requires that the father—or members of the group closely related through the patriline—contributes some resources to his offspring after weaning, and that the father's contribution increases relative to that of the mother.

**Figure 7:**
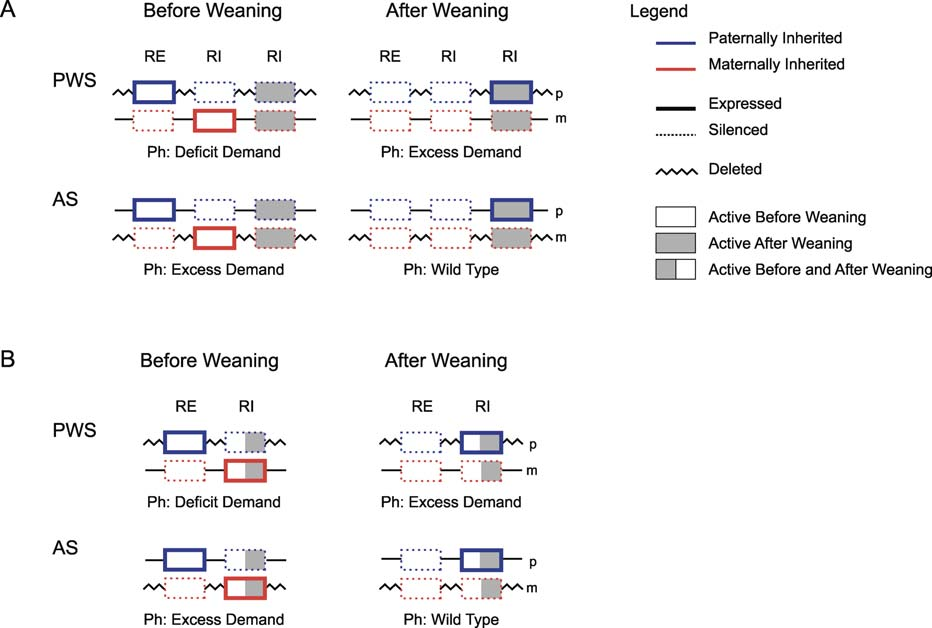
Composition of PWS/AS Cluster Each figure represents the composition in terms of type of gene (resource enhancer or resource inhibitor) and window of expression of each gene (either before weaning, or after weaning, or before and after weaning). Within each figure, I represent the genotype of a patient with PWS and AS. I use a zigzag line to represent a deleted strand. Underneath each scheme, I note the predicted phenotype. I assume that before weaning, the maternal contribution of resources (relative to the paternal one) is greater than σ̂#x0963; σ̂#x0302; but after weaning, the relative maternal contribution of resources is less than σ̂. I present two alternative compositions: (A) corresponds to the possibility that genes are expressed in each period (before or after weaning) but not across periods (before and after weaning). (B) corresponds to the possibility that genes are expressed across periods.

Consider the simplest scenario when mothers provide almost all the nutrients consumed by their offspring before weaning, but fathers contribute some nutrients afterward. In particular, assume that the relative contribution of fathers goes from σ^b^ > σ̂ before weaning to σ^a^ < σ̂ after weaning. According to the results presented in the previous section, a gene expressed before weaning is expected to be maternally silent if it is an RE but paternally silent if it is an RI. A gene expressed after weaning is expected to be paternally silent if it is an RE but maternally silent if it is an RI (Figure 7). A gene expressed before and after weaning that is able to modify its expression over time is expected to be maternally silent before weaning but paternally silent after weaning if it is an RE, and the other way around if it is an RI (Figure 7). The voice of the PI copy raises and eventually shouts out the MI copy's voice.

I propose that the PWS-AS cluster of imprinted genes contains either: (a) REs and RIs expressed before weaning but RIs expressed after weaning (Figure 7A); or (b) REs expressed before weaning but RIs expressed before and after weaning (Figure 7B). If this were the case, children with PWS would miss the expression of the REs before weaning and the RIs after weaning. The expected clinical phenotype is a deficit of appetite and weight before weaning and an excess of appetite and weight afterwards (Figure 7). Children with AS, on the other hand, would miss the expression of the RIs before weaning, but they would not miss the expression of any type of gene after weaning (Figure 7). The clinical phenotype expected would be an excess of appetite and weight before weaning, and normal appetite and weight afterward. These expectations match the clinical phenotype observed in PWS and AS.

## Discussion

The kinship theory of genomic imprinting was formulated under the assumption that only mammalian mothers invest in their offspring. Although biparental care is not common among mammals (less than 10% of genera), it is common in certain orders (close to 40% of genera in Perissodactyla, Carnivora, Rodentia, and Primates) [11] and more notably, observed in humans. Therefore, to understand the evolution of genomic imprinting as a result of intralocus conflict, the kinship theory should be extended beyond maternal contribution of resources only. In this research, I generalize the kinship theory by elaborating a model in which both parents can contribute any amount of resources, and maternal contribution only is a special case.

In the original formulation of the kinship theory, genomic imprinting evolves unless there is lifetime monogamy. The introduction of paternal care allows an alternative scenario when genomic imprinting does not evolve. If the cost experienced exclusively by the father due to the expression of a PI gene in the current offspring is equal to the cost experienced exclusively by the mother due to the expression of an MI gene in the current offspring, then deviations from an unimprinted expression are not favored by natural selection. An example to illustrate this point may shed light on what this condition entails. Consider a family in which the mother contributes most of the food, 75% (Figure 2). Assume that fathers (reproducing males) produce more offspring than mothers (reproducing females); notice that the average reproductive success of males does not have to be equal to the average reproductive success of females. While mothers conceive most of those offspring with the same father, 75% of them, the fraction of offspring that fathers conceive with the same mother represent 50% of the fathers' total production. Assume that the mother contributes food sources such as roots and fruits, and the father contributes meat. Hunting is more risky, 1.5 times more, than gathering food.

In this scenario, an additional contribution of resources to the current offspring reduces the amount of resources available to future offspring. The father provides 1/4 of the additional resources and suffers a cost four times the additional investment in the offspring. Half of the time, the cost experienced by the current father is not shared by the current mother. Hence the cost that the father incurs—due to the expression of a PI gene in the current offspring—that is not shared by the mother is (1 − κ*_M_*)*C_Pi_I_Px_* = (1/2) 4 (1/4) = (1/2). On the other hand, the mother provides 3/4 of the additional resources and suffers a cost that is 8/3 times the additional investment in the current offspring. One-fourth of the time, the cost experienced by the current mother is not shared by the current father. The cost the mother incurs—due to the expression of an MI gene in the current offspring—that is not shared by the father is (1 − κ*_P_*)*C_Mi_I_Mx_* = (1/4) (8/3) (3/4) = 1/2. Given that, in this case, (1 − κ*_M_*)*C_P_ =* (1 − κ*_P_*)*C_M_*, there is no intralocus conflict between the MI and the PI and genomic imprinting would not evolve.

In the original formulation of the kinship theory, there is only one direction of imprint for each kind of gene. If the gene considered is an RE, it is expected to be maternally silent. However, if the gene considered is an RI, it is expected to be paternally silent. The introduction of paternal care allows that the direction of the imprint is reversed for each kind of gene. Consider an RE, a gene is expected to be paternally silent if (1 − κ*_M_*)*C_P_ >* (1 − κ*_P_*)*C_M_*, but maternally silent if (1 − κ*_M_*)*C_P_ <* (1 − κ*_P_*)*C_M_*. Consider an RI, a gene is expected to be maternally silent if (1 − κ*_M_*)*B_P_ >* (1 − κ*_P_*)*B_M_*, but paternally silent if (1 − κ*_M_*)*B_P_ <* (1 − κ*_P_*)*B_M_*. Interestingly, I found that it is not necessary that fathers contribute more resources than mothers for this change in the direction of the imprint to happen.

If the cost experienced exclusively by the father due to his own investment is greater than the cost experienced exclusively by the mother due to her own investment, then an RE evolves to be paternally silent, even if the mother is contributing more resources than the father. I will return to the previous example to illustrate this condition. The cost the current father incurs—due to his own investment—that is not shared by the current mother is (1 − κ*_M_*)*C_Pi_* = (1/2) 4 = 2. The cost the current mother incurs—due to her own investment—that is not shared by the father is (1 − κ*_P_*)*C_Mi_* = (1/4) (8/3) = 2/3. Thus (1 − κ*_M_*)*C_Pi_* > (1 − κ*_P_*)*C_Mi_* and even if the mother contributes more than half the resources, an RE may still evolve to be paternally silent. In particular, if the mother contributes less than 75% of the resources, an RE evolves to be paternally silent. But if the mother contributes more than 75%, an RE evolves to be maternally silent.

The kinship theory requires genes expressed in the offspring that can affect the allocation of parental resources. During gestation, the interaction between mother and offspring is chemical—mediated by hormones and proteins, some of them secreted into the maternal bloodstream. During lactation, however, the interaction is mostly behavioral—mediated by actions performed by the offspring. In both cases, the relation between mother and offspring is intimate [48]. After weaning, the interaction between mother and offspring continues to be behavioral but becomes less intimate [48]. In general, genes expressed in the offspring can affect the allocation of maternal resources before birth by eliciting the segregation of hormones into the maternal bloodstream and after birth by eliciting behavioral acts that produce a maternal response. It is difficult to think how genes expressed in the offspring may influence the allocation of paternal resources during gestation. The only possibility would be that hormones secreted into the maternal blood stream elicit a maternal behavior that produces a paternal response. Before weaning, the contribution of food by the father is given indirectly through the mother. If a gene expressed in the offspring were to influence the allocation of paternal resources, it would have to elicit a behavior that produces a paternal provision of food to the mother who would be responsible for transferring such resources to the offspring. Furthermore the intimacy of the relation between mother and offspring would make mothers more likely to respond to any behavioral act of the offspring. After weaning, the food contribution by both parents is direct. Genes expressed in the offspring will have the same means and opportunities to influence each parent provision of nutrients.

From a taxonomic point of view, genomic imprinting was initially expected to be circumscribed to mammals and flowering plants. Such taxonomic distribution responds to the possibility of interaction between offspring and mother during gestation in eutherians and angiosperms. There is no room for interaction between mother and offspring during gestation in birds, most reptiles, and amphibians. Thus genomic imprinting was not expected to evolve in these classes. However it was soon noticed that after birth, the offspring can influence the allocation of maternal resources in a much more broad variety of organisms. The only condition is that there is extended postnatal parental care. Therefore imprinting is expected to be found in genes expressed after birth in mammals and after hatching in birds and fishes [48,49]. While biparental care is important in some mammalian orders, it is much more abundant in birds. The consideration of biparental care does change the pattern of imprint expected to evolve; in principle, it should not affect the expected taxonomic distribution of imprinting before and after birth. The only exception would occur in the particular case in which the life history and qualities of both parents as resource providers are equivalent, that is (1 − κ*_M_*)*C_P_ =* (1 − κ*_P_*)*C_M_*. In such case, genomic imprinting would not evolve.

The kinship theory, when extended to allow biparental care, provides a unique insight on the evolution of PWS and AS. I argue that the relative contribution of resources made by mammalian fathers increases after weaning, in particular that σ^b^ > σ̂ > σ^a^. If this is the case, an RE expressed before weaning is expected to be maternally silent, but another RE expressed after weaning is expected to be paternally silent. An RE expressed before and after weaning that can adjust its expression over time is expected to be maternally silent before weaning and paternally silent afterward (Figure 7). Similarly an RI expressed before weaning is expected to be paternally silent, but another RI expressed after weaning is expected to be maternally silent. An RI that is expressed before and after weaning is expected to be paternally silent before weaning and maternally silent afterwards (Figure 7). This explains that the loss of the PI copy of the PWS-AS cluster will result in a deficit of food intake and weight before weaning and an excess afterwards. The loss of the MI copy of this cluster will result in an excess of food intake before weaning and a normal food intake afterwards—if the genes expressed after weaning are RIs only. This matches the clinical phenotype of children with PWS and AS concerning appetite and weight. While the kinship theory can account for the aspects of PWS and AS concerning appetite and weight, there are other aspects of these disorders, mostly mental problems, that are difficult to interpret straight away.

Our understanding of the evolutionary forces and types of genes involved in the post-weaning phenotype of children with PWS might be important for the treatment of this disease. If the post-weaning phenotype is caused by an RI that is maternally silent, as I suggest, it could be possible to re-activate the maternal copy. However, it should be re-activated after weaning only and not before, because this would exacerbate the condition of children with PWS before weaning.

The phenomenon I am describing does not need genes that are expressed before and after weaning and thus change the direction of the imprint. It is enough to have a gene expressed before weaning and another one expressed afterwards. However my model does suggest the possibility that genes evolve to change the direction of the imprint from maternally silent to paternally silent or vice versa. I refer to these genes as flip-flop genes. While there is no evidence indicating that flip-flop genes may exist, I do not know of any attempt to find them. Interestingly, it is known that some genes can change the direction of the imprint depending on the tissue in which they end up being expressed. This is the case of gene *Grb10* in mouse, which is expressed from two promoters with opposite pattern of imprint, namely the paternal allele remains silent in most tissues but the maternal allele remains silent in the brain [50,51]. In both cases, the protein product is the same. If it is possible that a gene uses promoters that are imprinted in the opposite direction in different tissues, my prediction of imprinted genes using promoters that show the opposite pattern of imprint at different moments in developmental time does not seem too far fetched. If this were the case, it would be possible to observe a new type of mutation, one that fails to use the correct promoter in the correct developmental time. Such a mutation would result in complicated clinical phenotypes.

This analysis can be extended to other diseases in which imprinted genes are involved. Genes that affect the contribution of resources after weaning may not follow the standard pattern of expression (maternally silent REs and paternally silent RIs). PWS is unique because it is one of the few diseases caused by imprinted genes that affect post weaning growth. While Beckwith-Wiedemann and Silver-Russell syndromes are caused by imprinted genes, both involve growth disorders that manifest themselves during gestation and before weaning but not afterward. In this sense, PWS provides a unique insight into the forces acting on imprinted genes. However the characterization of the function of imprinted genes will allow us to find more genes that affect provision of parental resources after weaning. In this case, the role of fathers will become relevant.

## Methods

Consider a family formed by a mother, a father, and an offspring. Both parents' limited amount of resources can either be invested in the current offspring or kept for future ones. All individuals are diploid, and the population is at equilibrium. Let *x_m_* and *x_p_* be the level of expression of the maternally and paternally inherited copies of an allele in the current offspring, where *x_m_*, *x_p_* ≥0. The aggregate level of expression *x* = *x_m_* + *x_p_* determines the amount of maternal *i_M_* and paternal *i_P_* investment in the current offspring, *i_M_* = *g_M_*(*x*) and *i_P_* = *g_P_*(*x*) , and the residual investment of each parent in future offspring. For simplicity, I assume that the aggregate level of expression *x* determines the total amount of parental investment *i*, where *i* = *i_M_* + *i_P_* and *i* = *g*(*x*), and the investment of each parent is a constant fraction of the total investment, *i_M_* = σ*g*(*x*) and *i_P_* = (1 − σ)*g*(*x*). Such simplifying assumption ignores the scenario in which a gene expressed in the offspring can affect the contribution of maternal and paternal resources in the opposite sense; it is no longer possible that *di_M_*/*dx* > 0, *di_P_*/*dx* < 0 or *di_M_*/*dx* < 0, *di_P_*/*dx* > 0. This assumption is more plausible from a biological perspective and does not influence the model in any other way. Following the literature on genomic imprinting, I use the term resource enhancer (RE) to refer to a gene whose greater expression results in an enhanced allocation of parental resources, *di*/*dx* > 0; and resource inhibitor (RI) to refer to a gene whose greater expression results in a reduced allocation of parental resources, *di*/*dx* < 0.

Parental investment in the current offspring determines not only the fitness of the offspring *v_O_* (defined as the number of offspring left by the current offspring), *v_O_* = *h_O_*(*i_M_*, *i_P_*), but also the residual fitness of the mother *v_M_* and father *v_P_* (defined as the number of grand offspring, other than those produced by the current offspring, left by each of the parents of the current offspring), *v_M_* = *h_M_*(*i_M_*, *i_P_*) and *v_P_* = *h_P_*(*i_M_*, *i_P_*) . Consider a population fixed for a particular allele such that the aggregate level of expression at a loci homozygous for the resident allele is x*. Consider the fate of a rare epimutation that modifies the expression of the resident allele either when maternally inherited or when paternally inherited. The aggregate level of expression at a loci heterozygous for any of these mutants is *x*. Any change in expression can produce a change in fitness of the same *dv_O_*/*dx* > 0 or the opposite sign *dv_O_*/*dx* < 0. I use the term benefit *B* to refer to the first one and cost *C* to refer to the second one. If considering a RE the offspring derives a benefit at a cost to its parents. The offspring benefit is defined as the change in offspring fitness caused by a change in expression evaluated at x*, *B_O_* = *dv_O_*/*dx*|*_x_*_=x*_. The parental cost is the change in parental fitness caused by a change in expression evaluated at x*, *C*_Ω_ = −*dv*_Ω_/*dx|_x_*_=x*_ where Ω ∈{*M*,*P*}. The parental cost derived from a change in expression can be subdivided into two components: the cost derived from a greater investment, *C*_Ωi_ = ∂*v*_Ω_/∂*i*_Ω_, and the change in investment caused by a greater expression, −*I*_Ω*x*_ = *ι*_Ω_(*di*/*dx*)|*_x_*_=x*_, that is *C*_Ω_ = *C*_Ω*i*_ *I*_Ω*x*_. Notice that if considering an RI, it is the parents that derive a benefit, *B_M_* and *B_P_*, at a cost to their offspring *C_O_*.

Define κ*_P_* as a measurement of how change in maternal fitness affects paternal fitness, κ*_P_ = dv_P_/dv_M_*, and κ*_M_* as a measurement of how change in paternal fitness affects maternal fitness, κ*_M_ = dv_M_/dv_P_* where 0 ≤ κ*_M_*, κ*_P_* ≤ 1.

### Inclusive fitness.

The inclusive fitness of the MI copy in the current offspring *v_m_* is given by the number of offspring produced by the current offspring, plus the number of offspring produced by future maternal siblings weighed by the probability that the MI allele in the current offspring is present in future maternal siblings, *v_m_* = *v_O_* + ½*v_M_*. The inclusive fitness of the PI copy in the current offspring *v_p_* is given by the number of offspring produced by the current offspring, plus the number of offspring produced by future paternal siblings weighed by the probability that the PI allele in the current offspring is present in future maternal siblings, *v_p_* = *v_O_* + ½*v_P_*.

The inclusive fitness effect of the MI copy, *W_m_* = *dv_m_*/dx_m_|_x=x*_ = *dv_m_*/*dx*|_x=x*_, is:

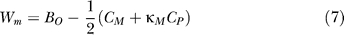
and the inclusive fitness effect of the PI copy, *W_p_* = *dv_p_*/dx_p_|_x=x*_ = *dv_p_*/*dx*|_x=x*_, is:

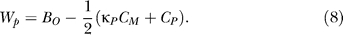



### Evolutionary stable level of expression.

An expression pattern of the MI and PI copies of an allele is evolutionarily stable 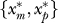 when adopted by most individuals in the population is resistant to invasion by any rare alternative strategy 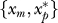 or 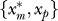 . In mathematical terms, this occurs when all the components of vector 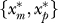 satisfy one of the following criteria [52]: (1) 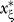 is a local fitness maximum, that is *W*_ξ_ = 0 and *∂W*_ξ_/∂x|_x=x*_<0, (2) 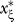 is a corner solution, that is 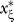 = 0 and *W*_ξ_ < 0 (where *ξ*∈{*m*,*p*}).

Imposing condition *W_m_* = 0 on Equation 7 yields the aggregate level of expression that is optimal from the perspective of the MI copy, 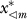 ,

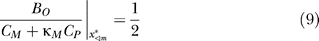
and imposing condition *W_p_* = 0 on Equation 8 yields the aggregate level of expression that is optimal from the perspective of the PI copy, 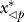 ,

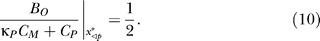



Consider that the initial condition is an equal expression of the MI and PI alleles 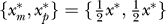 (unimprinted pattern of expression). When the optimal level of expression for the MI and PI alleles coincide, 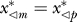 , there is no conflict. Selection does not favor deviations from the initial conditions, and the unimprinted pattern of expression is evolutionarily stable. When the optimal level of expression for the MI and the PI alleles diverge, 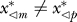 , there is conflict. Selection favors deviations from the unimprinted pattern of expression. If 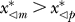 , the only pattern of expression that satisfies the conditions for evolutionary stability is 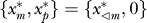 . If 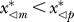 , however, this pattern of expression is 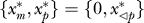 .

The difference

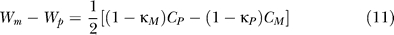
characterizes not only whether there is conflict between the MI and the PI copies but also, in case of conflict, what is the evolutionarily stable pattern of expression. Assume that for each function *W_ξ_* there is only one value 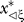 that satisfies condition *W_ξ_* = 0. If *W_m_* − *W_p_* = 0 then 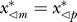 and there is no conflict. A positive sign, *W_m_* − *W_p_* > 0, implies that 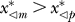 but a negative sign, *W_m_* − *W_p_* < 0, implies that 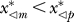 .

*No intragenomic conflict*: By imposing condition *W_m_* = *W_p_* on Equation 11, it is possible to conclude that there will be no conflict when:

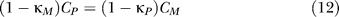



This condition is satisfied when one of the following is true: (1) κ*_M_* = κ*_P_* = 1; that is, changes in maternal fitness perfectly affect paternal fitness while changes in paternal fitness perfectly affect maternal fitness, for example in the case of lifetime true monogamy; (2) *C_M_* = *C_P_* = 0 where *C_M_* = *C_Mi_I_Mx_* and *C_P_* = *C_Pi_I_Px_*; (2a) *I_Mx_* = *I_Px_*= 0, that is, the expression of the gene considered does not influence parental investment; (2b) *I_Mx_* = 0 and *C_Pi_* = 0; (2c) *C_Mi_* = 0 and *I_Px_* = 0, that is, the expression of the gene considered does not influence the investment of one of the parents while the investment of the other father is cheap, meaning that its investment does not translate in any cost for the provider; (2d) *C_Mi_* = *C_Px_* = 0 investment is cheap for both parents; (3) (1 − κ*_M_*)*C_P_* = (1 − κ*_P_*)*C_M_*, that is, the fraction of paternal cost caused by paternal investment that does not translate into maternal cost, has to be equal to the fraction of maternal cost caused by maternal investment that does not translate into paternal cost.

Substituting *C_M_* = *C_Mi_I_Mx_* and *C_P_* = *C_Pi_I_Px_* in Equation 12 and keeping in mind that *I_Mx_* = σ (∂*g*/∂*x*) and *I_Px_* = (1 − σ) (∂*g*/∂*x*), it is possible to determine the fraction of the total resources contributed by the mother, σ, which extinguishes the conflict:

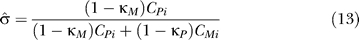



Such a point exists as long as the fitness functions are continuously differentiable. If σ = σ̂, genomic imprinting does not evolve.

It is worth noticing that the conflict's extinction does not require both parents to contribute the same amount of resources. In particular, if the cost of paternal investment experienced by the father but not shared with the mother is greater than the cost of maternal investment experienced by the mother but not shared with the father, (1 − κ*_M_*)*C_Pi_* > (1 − κ*_P_*)*C_Mi_*, then the extinction of the conflict happens for a value σ such that mothers contribute more resources than fathers, σ̂ > ½, and vice versa. This might be relevant in nature because mothers contribute more resources than fathers in many cases.

Intragenomic conflict: If *W_m_* ≠ *W_p_* there is conflict between the MI and PI copies of an RE and genomic imprinting evolves. If (1 − κ*_M_*)*C_P_* > (1 − κ*_P_*)*C_M_* then *W_m_* > *W_p_* and the pattern of expression 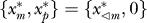 is the only evolutionarily stable stategy (ESS). If (1 − κ*_M_*)*C_P_* < (1 − κ*_P_*)*C_M_*, however, then *W_m_* < *W_p_* and the pattern of expression 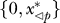 is the only ESS.

Similarly, if σ ≠ σ̂, there is conflict between the MI and PI copies of an RE and genomic imprinting evolves. If σ < σ̂, then 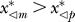 , and the pattern of expression 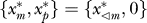 is the only ESS. If σ > σ̂, then 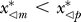 , and the pattern of expression 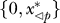 is the only ESS.

Even if the fitness functions are not continuous in σ̂, this value still marks the boundary between the two patterns of expression, 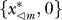 and 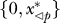 . This can be concluded from Equation 11 by noticing that for an RE, if there were maternal care only σ = 1, then 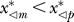 . However, if there were paternal care only σ = 0 then 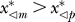 .

If the gene considered were an RI, then *W_m_* − *W_p_*= ½ [(1 − κ*_P_*)*B_M_* − (1 − κ*_M_*)*B_P_*] and the expected patterns of expression are the opposite to the RE ones. If (1 − κ*_M_*)*B_P_* > (1 − κ*_P_*)*B_M_*, then *W_m_* < *W_p_* and the pattern of expression 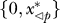 is the only ESS. If (1 − κ*_M_*)*B_P_* < (1 − κ*_P_*)*B_M_*, however, then *W_m_* > *W_p_* and the pattern of expression 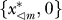 is the only ESS. Similarly, if σ < σ̂ then 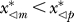 and the pattern of expression 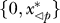 is the only ESS. If σ > σ̂ then 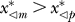 and the pattern of expression 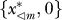 is the only ESS.

### Parental contribution changes over time.

All previous results apply when, given a level of expression, parental investment does not change over the window of time when the gene is expressed. This might be the case either because parental investment does not change over developmental time altogether or because parental investment does change, but not within the window of time when the gene is expressed. In this section, I will discuss the case when, for the same level of expression, each parent invest more or less at different developmental stages.

Inclusive fitness: Let *t* represent time in the life of the current offspring, and [0,*T*]_g_ be the window of time when gene *g* is expressed. The relative investment of each parent is a function of time, σ = *f_M_*(*t*); therefore *i*_Ω_ = *f*_Ω_(*t*)*g*(*x*). The inclusive fitness effect of the MI allele when the gene is expressed within the window of time [0,*T*] is:

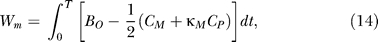
and the inclusive fitness effect of the PI allele is:

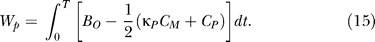



For simplicity, I consider that parental investment may differ between two periods of time, namely, before weaning *t* < *t^w^* and after weaning *t* > *t^w^*. Given a certain level of expression *x*, the amount of resources contributed by each parent remains constant within each period but may change between periods, that is *i_M_* = σ*^b^g*(*x*) for *t* ≤ *t^w^* but *i_M_* = σ*^a^g*(*x*) for *t* > *t^w^*.

*Evolutionary stable level of expression*: The conditions for evolutionary stability are the conditions provided in the previous section; however, now the vector considered contains two vectors—one for the pattern of expression before weaning and another one for the pattern of expression afterwards. Which pattern of expression evolves depends on whether the expression of the gene considered can be modified during each period of time.

*Fixed expression:* Assume that the gene's level of expression is unable to change through developmental time. Let μ = *t^w^*/*T* be the fraction of time before weaning within the window of time in which the gene considered is expressed. There is no conflict between the MI and PI alleles whenever *σ^ba^* = σ̂, where σ*^ba^ = μ*σ*^b^ +* (1−*μ*)σ*^a^* is the mean maternal investment over the window of time when the gene considered is expressed. Intragenomic conflict occurs when *σ^ba^* ≠ σ̂. Consider an RE. If *σ^ba^*< σ̂ then 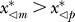 and the pattern of expression 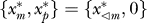 is the only ESS. If *σ^ba^* > σ̂ then 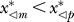 and the pattern of expression 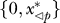 is the only ESS. Therefore, whether a gene evolves pattern 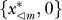 or 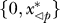 depends on whether the window of time when the gene considered is expressed occurs mostly before or after weaning.

*Variable expression*: Assume that the level of expression of the gene considered can be adjusted over developmental time. No conflict exists between the MI and PI alleles whenever *σ^b^* = *σ^a^* = σ̂. Let the two ordered pairs 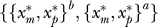 represent the level of expression of the MI and PI copies before and after weaning respectively. There is intragenomic conflict when either σ^*b*^ ≠ σ̂ or σ^*a*^ ≠ σ̂ or both. Consider an RE. If σ^*b*^ > σ̂ and σ^*b*^ < σ̂ then before weaning 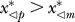 but after weaning 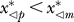 , and the pattern of expression 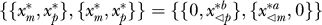 is the only ESS. If σ^*b*^ < σ̂and σ^*a*^ > σ̂ then before weaning 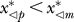 but after weaning 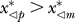 , and the pattern of expression 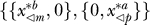 is the only ESS. These kinds of genes will show two different patterns of expression before and after weaning.

## Abbreviations

AS: Angelman syndrome
MI: maternally inherited
PI: paternally inherited
PWS: Prader-Willi syndrome
RE: resource enhancer
RI: resource inhibitor
